# Artificial intelligence in predicting macular hole surgery outcomes: a focus on optical coherence tomography parameters

**DOI:** 10.1186/s12886-025-04256-9

**Published:** 2025-07-22

**Authors:** Yucel Ozturk, Abdullah Ağın, Burcu Yelmi, Nilufer Zorlutuna Kaymak

**Affiliations:** 1 Department of Ophthalmology, Faculty of Medicine, Istanbul Health and Technology University, Istanbul, Turkey; 2 Department of Ophthalmology, University of Health Sciences, Haseki Training and Research Hospital, Fatih, Istanbul, 34130 Turkey; 3 World Eye Hospital, Istanbul, Turkey; 4 Department of Ophthalmology, University of Health Sciences, Kartal Dr. Lutfi Kirdar City Hospital, Istanbul, Turkey

**Keywords:** Macular hole, Artificial intelligence, GPT, THI, MHI, HFF, BHD

## Abstract

**Purpose:**

To evaluate the predictive performance of optical coherence tomography (OCT)-based indices and artificial intelligence (AI) using a Generative Pre-Trained Transformer (GPT) model and compare them with traditional logistic regression in forecasting anatomical success following macular hole (MH) surgery.

**Methods:**

This retrospective observational study included 51 eyes of 51 patients who underwent pars plana vitrectomy for idiopathic MH. Preoperative OCT measurements of macular hole index (MHI), traction hole index (THI), hole form factor (HFF), basal hole diameter (BHD), and minimum hole diameter (MHD) were recorded. GPT-based AI predictions were generated using masked input data. A logistic regression model was developed with the same variables. Predictive performance was assessed using accuracy, area under the curve (AUC), positive predictive value (POPV), negative predictive value (NPV), and Kappa statistics.

**Results:**

Anatomical success was achieved in 72.5% of cases. MHI, THI, and HFF were significantly higher in the successful group (*p* < 0.0001). GPT achieved an accuracy of 77.0% and AUC of 0.770, with perfect POPV (1.000) but low NPV (0.452). Logistic regression outperformed GPT, achieving an accuracy of 84.3%, an AUC of 0.759, a higher NPV (0.800), and better agreement (Kappa 0.568 vs. 0.392). BHD and MHD showed poor predictive power (AUC 0.291).

**Conclusion:**

OCT-derived indices, especially MHI, THI, and HFF, effectively predict MH surgery outcomes. Logistic regression based on actual patient data demonstrated superior predictive performance compared to GPT. AI models hold potential but require further development, integration of multimodal data, and validation before clinical application.

## Introduction

The role of artificial intelligence (AI) in healthcare is pivotal, as it significantly aids in clinical decision-making and case management. Its potential to diagnose a wide range of eye conditions is a promising development [1].

The Generative Pre-Trained Transformer (GPT), a creation of OpenAI (San Francisco, CA, USA), has shown significant potential in healthcare diagnosis. These models can be trained to produce appropriate responses, with GPT 4.0 currently standing as the most advanced version [2–4].

A macular hole (MH), a round opening in the center of the fovea, is a treatable cause of central vision loss. The use of optical coherence tomography (OCT) is crucial in recognizing and evaluating an MH, and it plays a significant role in increasing surgical success with new techniques [5–9].

Various OCT parameters have been used to predict outcomes after MH repair. These measurements include the minimum distance between hole margins, maximum diameter at the top of the hole, longest base diameter, and MH height. Using these OCT measurements, various indices have been developed to predict outcomes, including the hole form factor (HFF), MH index (MHI), and traction hole index (THI) [10–13].

As AI becomes increasingly integrated into medical practice, its role in ophthalmology remains a subject of growing interest. By assessing AI’s predictive capabilities in comparison with established clinical tools, this study contributes to the ongoing efforts to enhance decision-making in MH management. The potential of AI in predicting postoperative success in MH surgery is a promising development. Future advancements in AI, particularly in combination with traditional methodologies, may lead to improved preoperative evaluation and personalized surgical planning. In our study, we aimed to investigate the potential of AI in predicting postoperative success in MH surgery. By utilizing preoperative OCT parameters and indices, we explored how AI-based models, particularly GPT, could assist in preoperative assessment. Additionally, we incorporated logistic regression to compare AI-generated predictions with a traditional statistical approach, ensuring a more comprehensive evaluation of predictive accuracy.

## Methods

This is a comprehensive retrospective observational study. Fifty-one eyes of 51 patients who underwent 25-gauge pars plana vitrectomy with the diagnosis of idiopathic MH in the ophthalmology clinics between 2022 and 2024 were included in the study.

Patients with idiopathic MHs who underwent pars plana vitrectomy (PPV) and internal limiting membrane peeling surgery were included in this study. Completing at least 6 months of follow-up after surgery was one of the inclusion criteria. Exclusion criteria included retinopathy due to DM or HT, history of retinal detachment surgery, high myopia (axial length greater than 26.00 mm or refractive error greater than −6.00D), history of uveitis or trauma, fundus disease other than MH, history of intravitreal injection.

A detailed clinical evaluation confirmed the diagnosis of an MH. We meticulously collected the demographic and clinical data of the patients, ensuring the reliability of our study. The best corrected visual acuity (BCVA) was recorded using the Snellen chart before surgery, and at the last follow-up, it was converted to logMAR. All patients included in the study underwent comprehensive ophthalmologic evaluations, including slit-lamp biomicroscopy, dilated fundus examination, and OCT. Postoperatively, patients were divided into two groups: successful (group 1) and unsuccessful (group 2). Postoperative macular OCT was evaluated as successful if the hole was anatomically closed and unsuccessful if it was not closed.

The surgical procedure was performed by the same surgeon (YO) using a 3-port 25-gauge pars plana vitrectomy technique. In patients with cataracts, the surgery was performed as a combined phacovitrectomy. Twenty-five-gauge trocar cannulas were placed approximately 3.5 mm behind the limbus. During surgery, after the core vitrectomy was completed, the posterior hyaloid was assessed for separation, and if not, it was carefully separated from the retina. Brilliant blue dye was used to visualize and stain the internal limiting membrane (ILM) in the macular region, and then the ILM was meticulously peeled off. After the fluid-air exchange, the air in the vitreous cavity was replaced with 20% sulfur hexafluoride (SF6) gas. After the surgery, the patients were asked to lie in the prone position for 1 week.

Spectralis SD-OCT (Spectralis; Heidelberg Engineering, Heidelberg, Germany) was used for OCT imaging. Spectralis SD-OCT incorporates real-time eye-tracking software and has the advantage of improving quality and segmentation accuracy in the macular region. OCTs from all visits (before MH formation, during MH formation, and after vitrectomy) were examined. Macular scans were performed using 25 horizontal B-scans focusing on the fovea. The area to be measured was taken from the 12th or 13th image, which was precisely centered on the macula. The caliper function in the device's software was used for measurement. Images with an image quality score above 20 were used in patients without cataracts. The lowest quality score was determined to be 15 in patients with cataracts. Images with lower quality scores were not taken into consideration. The baseline MH size, minimum MH size, hole height, and right and left arm length were measured in OCT images using the device's internal measurement function. According to the measurements, previously reported MHI, HFF, and THI were calculated. MHI was defined as the ratio of MH height to the diameter of the base of the hole, while THI was defined as the ratio of the maximum height to the narrowest diameter of the MH. HFF was calculated by summing the nasal and temporal limb lengths and dividing by the longest base diameter. Measurements were made by two different observers (YO, BY) at different times. Each measurement was measured twice by each observer and averaged. The average of the measurements of the two observers was then used for statistical analysis. To assess the repeatability of the measurements before analysis, inter-observer and intra-observer intraclass correlation coefficients (CC) were determined using 30 randomly selected images. The intra-observer CC value was > 0.9, and the inter-observer CC value was > 0.9.

ChatGPT (version 4.0) was asked various questions about the definition and treatment of MHs to test the knowledge of this GPT bot (Table 1). The answers were evaluated by two vitreoretinal surgeons (YO, AA). The answers given by ChatGPT were found to be consistent with the literature. Then, detailed questions were asked about OCT markers and MHI, HFF, and THI indices used to predict the surgical outcome of MHs, and the formulas of these indices were requested (Table 2). ChatGPT's formulas and explanations are presented in Tables 1 and 2. After deciding that the descriptive information given by ChatGPT was sufficient, we masked the names of the patients and their surgical outcomes. We provided them with the preoperative minimum hole diameter (MHD), baseline hole diameter (BHD) OCT measurements, and MHI, TFF, and HFF index values ​​for their interpretation. ChatGPT was asked to predict surgical outcomes separately for each measurement and index. The prediction results were compared with the actual results. Predictions were made on a per-case basis, with each patient's data entered and evaluated individually. The input dataset was fully masked, including only de-identified preoperative variables such as MHD, BHD, MHI, THI, and HFF. Neither patient identifiers nor surgical outcomes were provided during the prediction process, ensuring unbiased input handling by the GPT model.

**Table 1:** ChatGPT’s answers and indexes regarding macular hole

The macular hole is a small break or defect in the central part of the retina, known as the macula, which is responsible for sharp, central vision. The macula is critical for tasks like reading, driving, and recognizing faces. A macular hole disrupts this part of the retina, leading to blurred or distorted central vision.
Causes:
• Aging: The most common cause is the age-related shrinking of the vitreous gel (a jelly-like substance in the eye) that pulls on the macula.
• Trauma: Eye injuries can also cause macular holes.
• High Myopia (Nearsightedness): Individuals with severe myopia are at greater risk.
• Retinal Disorders: Conditions like epiretinal membrane or diabetic retinopathy can contribute.
• Macular Edema: Fluid buildup in the macula can weaken its tissue, increasing the likelihood of a hole.
Symptoms:
• Blurred or distorted central vision.
• Difficulty with tasks requiring fine detail, like reading.
• A central dark or blank spot in vision.
• Lines or objects appearing bent or wavy (metamorphopsia).
Diagnosis:
Macular holes are diagnosed through:
• Ophthalmoscopy: Direct visualization of the retina.
• Optical Coherence Tomography (OCT): A detailed imaging technique that provides cross-sectional images of the retina to confirm the presence and size of the hole.
Treatment:
• Observation: Small holes may resolve on their own, especially in the early stages.
• Vitrectomy Surgery: This is the most common treatment. In this procedure, the vitreous is removed, and a gas or air bubble is inserted to help the macula heal.
• Post-Surgery: Patients often need to maintain a face-down position for days to keep the gas bubble in place.

**Table 2:** Formulas and explanations of the metrics

1. MHI (Macular Hole Index)
Formula: MHI = Macular Hole Height/Minimum Hole Diameter
Explanation: MHI is the ratio of the macular hole height to the minimum hole diameter. This ratio is used to predict the probability of macular hole closure. Generally, MHI values ​​of 0.5 and above increase the likelihood of closure.
2. THI (Tractional Hole Index)
Formula: THI = Macular Hole Height/Basal Hole Diameter
Explanation: THI is the ratio of the macular hole height to the basal hole diameter. It shows the effect of tractional forces. A THI of 0.6 and above increases the probability of closure.
3. HFF (Hole Form Factor)
Formula: HFF = (c + d)/a
Explanation: HFF is calculated by dividing the basal diameter (a) by the sum of the lengths of the left arm (c) and right arm (d) located at the top of the hole. HFF values ​​of 0.9 and above may increase the probability of successful closure.

The obtained data were analyzed using SPSS 22.0 (SPSS Inc., Chicago, IL, USA). Data distribution was assessed using the Shapiro–Wilk test. As some parameters did not conform to a normal distribution, non-parametric tests were employed. The Mann–Whitney U test was used to compare median values between groups. To evaluate the predictive performance of AI-generated classifications, Cohen's Kappa was calculated to assess the agreement between predicted and actual outcomes. Receiver Operating Characteristic (ROC) curves were generated to determine the discriminative ability of predictive indices, with the Area Under the Curve (AUC) used as a measure of accuracy. Additionally, a confusion matrix was constructed to analyze classification performance, including True Positive (TP), True Negative (TN), False Positive (FP), and False Negative (FN) rates. Positive Predictive Value (POPV) and Negative Predictive Value (NPV) were also computed to assess model validity further. Furthermore, a logistic regression model was developed using actual patient data to establish a benchmark for predictive accuracy. The model was trained on preoperative OCT parameters and indices (MHI, THI, HFF, BHD, and MHD) as independent variables, with MH closure as the dependent variable (0: non-closure, 1: closure). This statistical approach enabled an objective comparison between AI-generated predictions and actual clinical data, ensuring a robust assessment of GPT’s predictive performance. The predictive capabilities of logistic regression and AI were compared using accuracy, AUC, POPV, NPV, and Kappa statistics. A *p*-value below 0.05 was considered statistically significant.

## Results

**Fig. 1:**
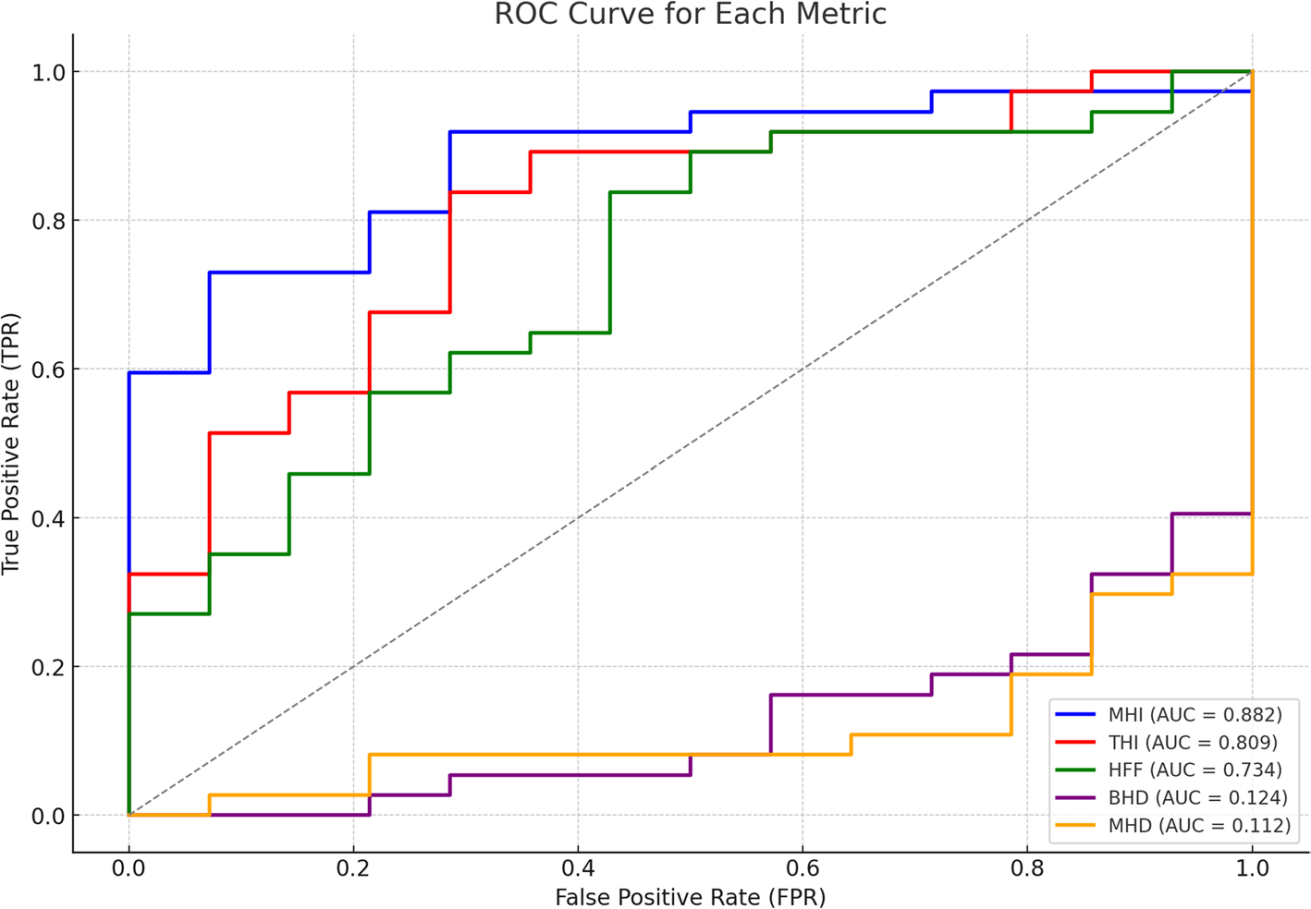
ROC curve for each metric: ROC curves demonstrating the performance of different OCT-derived metrics in predicting macular hole surgery outcomes

**Fig. 2:**
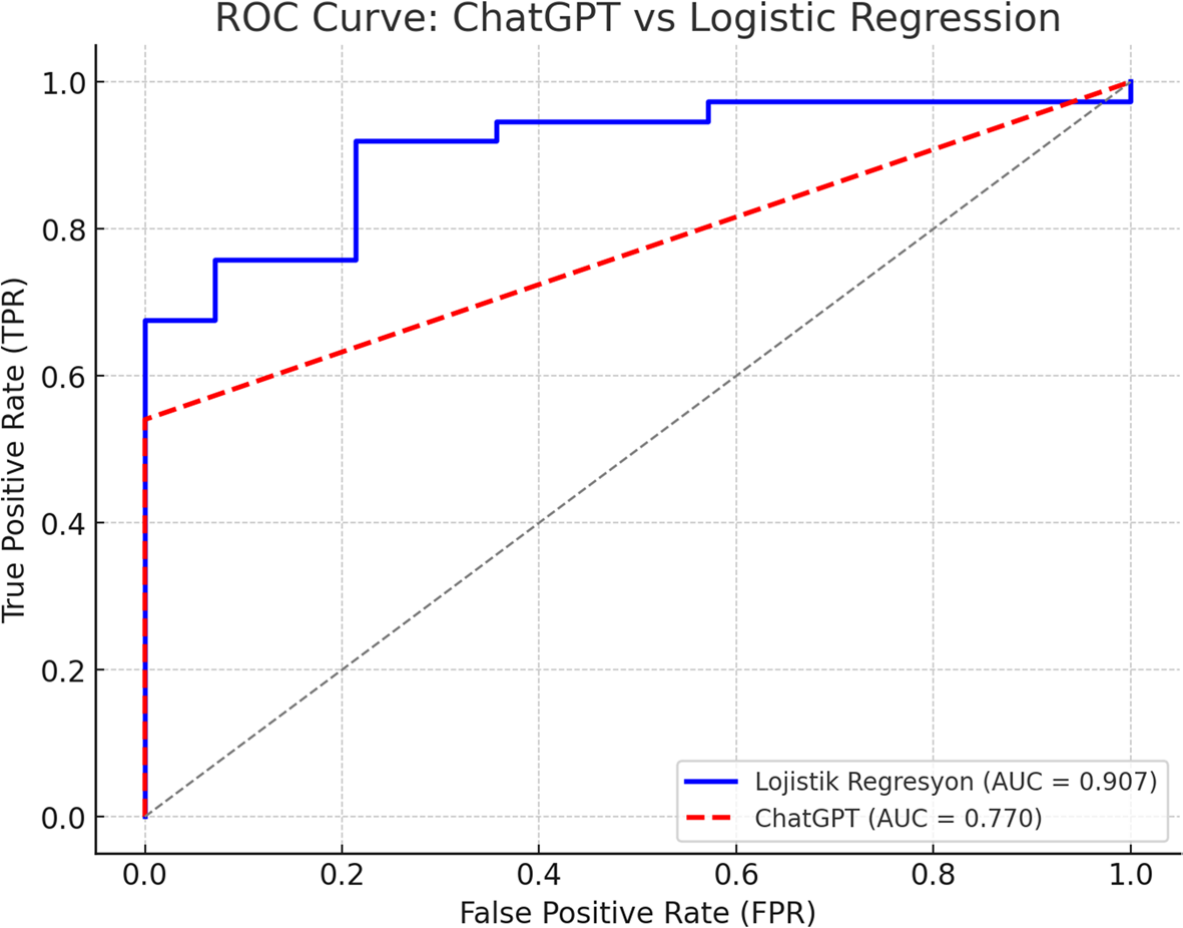
ROC curve: ChatGPT vs logistic regression: comparison of ROC curves between a logistic regression model and ChatGPT in predicting surgical outcomes of MH using preoperative OCT parameters

Fifty-one eyes of 51 patients were included in the study. The mean age was 67.7 ± 6.7 (59–91) years, and the male-to-female ratio was 20:31. As a result of MH surgery, there were 37 (72.5%) patients in group 1 and 14 (27.5%) in group 2. While the mean preoperative BCVA was 1.5 ± 0.4 logarithm of the minimum angle of resolution (logMAR), the mean BCVA at the last follow-up after surgery was 0.8 ± 0.3 logMAR (*p* < 0.001). Table 3 presents the mean, standard deviation, and *p*-values for five key metrics: MHI, THI, HFF, BHD, and MHD. The results indicate that Group 1 exhibits significantly higher mean values for MHI (0.564 vs. 0.344, *p* < 0.0001), THI (1.809 vs. 0.816, *p* < 0.0001), and HFF (0.844 vs. 0.678, *p* < 0.0001). Similarly, BHD and MHD were significantly larger in Group 2 (*p* < 0.0001). The confusion matrix results (Table 4) provide insights into the predictive accuracy of these indices. MHI demonstrated a TP rate of 39.22% and a FN rate of 33.33%. THI had the highest TP rate at 66.67% but also showed a 19.61% FP rate. HFF performed comparably, with a TP rate of 41.18% and an FN rate of 31.37%. In contrast, BHD and MHD showed very low TP rates (5.88%) and high FN rates (66.67%) (Table 4). Table 5 further refines these findings by analyzing the area under the curve (AUC), POPV, NPV, and Kappa agreement scores. MHI had the highest AUC (0.770) and perfect POPV (1.000), though its NPV remained low at 0.452. THI and HFF followed with AUC values of 0.602 and 0.677, respectively, and moderate POPV and NPV scores. BHD and MHD, however, exhibited poor discriminatory ability, both with an AUC of 0.291 and negative Kappa values, reinforcing their limited predictive power (Table 5 and Fig. 1). ChatGPT demonstrated a perfect POPV  (1.000); however, the logistic regression model outperformed it in terms of overall accuracy (0.843 vs. 0.770), NPV (0.800 vs. 0.452), and Cohen’s Kappa (0.568 vs. 0.392), indicating greater predictive reliability (Table 6 and Fig. 2).

**Table 3:** Comparison of the metrics between the groups

	**Group 1**		**Group 2**		
Metric	**Mean**	**Std Dev**	**Mean**	**Std Dev**	***p*-value**
MHI	0.564	0.203	0.344	0.081	< 0.0001
THI	1.809	1.611	0.816	0.346	< 0.0001
HFF	0.844	0.190	0.678	0.162	< 0.0001
BHD	977.892	342.554	1569.786	478.478	< 0.0001
MHD	400.622	196.000	683.929	154.123	< 0.0001

**Table 4:** Confusion matrix results (Percentages)

**Metric**	TP (%)	TN (%)	FP (%)	FN (%)
MHI	39.22	27.45	0.00	33.33
THI	66.67	7.84	19.61	5.88
HFF	41.18	21.57	5.88	31.37
BHD	5.88	13.73	13.73	66.67
MHD	5.88	13.73	13.73	66.67

**Table 5:** Combined results: AUC, predictive values, and agreement

Metric	AUC	POPV	NPV	Kappa
MHI	0.770	1.000	0.452	0.392
THI	0.602	0.773	0.571	0.242
HFF	0.677	0.875	0.407	0.274
BHD	0.291	0.300	0.171	−0.262
MHD	0.291	0.300	0.171	−0.262

**Table 6:** ChatGPT vs. logistic regression model comparison

Model	Accuracy	AUC	POPV	NPV	Kappa
Logistic Regression	0.843	0.759	0.854	0.800	0.568
ChatGPT	0.770	0.770	1.000	0.452	0.392

## Discussion

This study highlights the predictive value of OCT-derived indices in determining the anatomical success of MH surgery. It compares the performance of artificial intelligence (AI) modeling with traditional statistical methods. Consistent with the literature, our analysis confirmed that MHI, THI, and HFF were the most effective parameters for predicting surgical outcomes. MHI demonstrated the highest predictive capacity (AUC: 0.770, POPV: 1.000), reinforcing its role as a reliable marker of successful MH closure. However, its relatively low NPV (0.452) signals that MHI alone may not sufficiently identify non-closure cases, warranting caution when used in isolation. THI and HFF also showed moderate predictive power (AUC: 0.602 and 0.677, respectively), suggesting these indices may be better utilized in combination rather than alone. In contrast, BHD and MHD, although traditionally considered necessary, exhibited poor predictive capacity (AUC: 0.291) and negative Kappa values. These values suggest a lack of agreement between predicted and observed outcomes, highlighting that BHD and MHD may not contribute meaningfully to reliable preoperative prediction models. These findings align with newer studies suggesting that simple linear measurements may not sufficiently represent the complex anatomical and biomechanical characteristics influencing surgical success. The confusion matrix analysis further supported these findings. MHI and THI demonstrated true favorable rates (39.22% and 66.67%, respectively), while BHD and MHD showed very low TP (5.88%) and high FN (66.67%), underscoring their limited utility. THI's FP rate (19.61%) also raises concerns about potential overestimation of closure probability, highlighting the importance of multiparametric evaluation.

The growing role of AI, specifically GPT-based models, in clinical decision support was another key aspect of this study. While GPT demonstrated reasonable predictive performance (accuracy 0.770, AUC 0.770), it was surpassed by the logistic regression model, which achieved higher accuracy (0.843), better NPV (0.800), and superior agreement (Kappa 0.568 vs. 0.392). GPT's perfect POPV (1.000) suggests it effectively predicts successful closures but may inadequately identify non-closures, limiting its standalone clinical utility. While the AUC values of GPT and logistic regression were similar (0.770 vs. 0.759), this should not be interpreted as equal clinical performance. Logistic regression demonstrated substantially better agreement (Kappa 0.568 vs. 0.392) and a markedly higher NPV (0.800 vs. 0.452), indicating superior reliability in identifying non-closure cases. Clinically, this distinction is critical, as missing a non-closure case could lead to delayed or suboptimal management. This interpretation is consistent with recent findings highlighting the limitations of single-modality predictors and the importance of model sensitivity in preoperative planning [14]. Meanwhile, these findings emphasize that while AI is promising, its current iteration remains inferior to traditional statistical methods in this specific context. Our findings align with several recent publications exploring the prognostic power of OCT indices. Kumar et al. [14] confirmed that higher MHI and HFF correlate positively with visual acuity, whereas BHD negatively correlates, mirroring our results. Similarly, Chen and Yang [15] highlighted the impact of MHI and THI on surgical success in persistent MHs managed with secondary gas injections. Their findings reinforce the relevance of biomechanical indices in predicting outcomes. Beyond linear indices, recent studies propose novel parameters enhancing predictive models. Ozturk et al. [16] introduced the Cyst Hole Area Index (CHAI), which considers cystic configurations surrounding the MH and shows a strong negative correlation with postoperative visual acuity. This suggests that models incorporating morphological complexity could offer superior prediction accuracy. Furthermore, anatomical closure does not guarantee functional recovery. The integrity of the outer retinal layers, particularly the photoreceptor complex, plays a critical role in determining visual outcomes. Yang et al. demonstrated that smaller ellipsoid zone (EZ) disruption diameters and higher EZ reflectivity strongly correlate with better postoperative BCVA. Likewise, Patel et al. [17] proposed the “indistinct retinal outer layers (I-ROL)” marker, showing that a greater preoperative extent of I-ROL predicts better postoperative external limiting membrane (ELM) restoration and visual recovery. Supporting this, Bajdik et al. [18] developed the A/B index, incorporating ELM-GCL distance. Their model outperformed MHI in predicting long-term visual acuity, emphasizing that future predictive tools should prioritize photoreceptor and retinal layer assessments alongside geometric parameters.

Choroidal microvascular health also influences visual prognosis. Chen et al. [19] reported that increased choroidal blood flow density and favorable changes in foveal avascular zones correlate with better postoperative BCVA. This underscores the need for integrating vascular assessments into predictive models, as vascular status may impact photoreceptor survival and retinal healing post-surgery.

The AI analysis in our study adds to the growing discourse on machine learning applications in ophthalmology. Although GPT showed potential, its reliance on limited OCT inputs constrains its performance. As An et al. [20] suggesting, incorporating complex three-dimensional OCT data, indices like the MH Outer Index (MHOI), and clinical variables such as preoperative BCVA or ILM peeling parameters, significantly enhances prediction models. This approach paves the way for AI systems trained on multimodal datasets, improving accuracy and clinical relevance.

One of the most significant strengths of this study is the direct comparison between AI-generated predictions and a traditional logistic regression model trained on actual patient data. Unlike GPT, which relies solely on predefined input values without contextual clinical understanding, the logistic regression model incorporates accurate preoperative OCT parameters and clinical outcomes, allowing for more robust and data-driven predictive performance. Our analysis revealed that the logistic regression model achieved higher predictive accuracy (84.3% vs. 77.0%), a superior NPV (0.800 vs. 0.452), and better agreement (Kappa 0.568 vs. 0.392) compared to GPT. These results underscore the advantage of statistically validated models that leverage accurate clinical datasets over AI models that operate without understanding complex patient-specific variables. While GPT demonstrated high specificity with a perfect POPV (1.000), it struggled in identifying negative cases, likely due to its inability to incorporate nuanced clinical factors and variability in anatomical presentations. This comparative analysis highlights that, at least in its current form, AI models like GPT should serve as supportive tools rather than standalone predictors in surgical planning. Logistic regression, grounded in real-world data, remains more reliable for outcome prediction in MH surgery. Future AI models must integrate larger, multimodal datasets, including clinical variables, volumetric OCT data, and microvascular information, to truly match or surpass the performance of traditional statistical approaches.

Nevertheless, this study has limitations. Its retrospective design introduces potential selection bias, and the relatively small sample size may limit generalizability. Additionally, while OCT indices provide valuable anatomical insights, they overlook patient-specific variables such as age, axial length, symptom duration, and preoperative visual acuity, all known to influence outcomes. Future predictive models must adopt a holistic approach, integrating multimodal imaging data (OCT, OCTA), volumetric analyses, and comprehensive patient demographics.

Another critical consideration is the interpretability and clinical integration of AI models. GPT's lower NPV and moderate accuracy highlight the necessity of clinician oversight when deploying AI tools in surgical planning. AI should complement, not replace, clinical expertise, assisting in decision-making while accounting for nuanced factors beyond the model’s dataset.

In conclusion, this study reaffirms the predictive value of OCT-derived indices, particularly MHI, THI, and HFF, in forecasting anatomical success after MH surgery. It also emphasizes that simple structural measurements like BHD and MHD may not adequately capture the complexity of MH pathophysiology. Incorporating assessments of photoreceptor integrity, chorioretinal microvasculature, and patient-specific factors will likely enhance predictive accuracy. AI models show promise but require further refinement and validation. Future studies should focus on integrating multimodal datasets and expanding sample sizes to develop robust, clinically applicable predictive tools that support personalized surgical planning and improve patient outcomes.

## Data Availability

The datasets generated and/or analyzed during the current study are available from the corresponding author on reasonable request.
